# Induced superconductivity in high-mobility two-dimensional electron gas in gallium arsenide heterostructures

**DOI:** 10.1038/ncomms8426

**Published:** 2015-06-11

**Authors:** Zhong Wan, Aleksandr Kazakov, Michael J. Manfra, Loren N. Pfeiffer, Ken W. West, Leonid P. Rokhinson

**Affiliations:** 1 Department of Physics and Astronomy, Purdue University, West Lafayette, Indiana 47907, USA; 2 Department of Electrical Engineering, Purdue University, West Lafayette, Indiana 47907, USA; 3 Department of Materials Engineering, Purdue University, West Lafayette, Indiana 47907, USA; 4 Birck Nanotechnology Center, Purdue University, West Lafayette, Indiana 47907, USA; 5 Department of Electrical Engineering, Princeton University, Princeton, New Jersey 08544, USA

## Abstract

Search for Majorana fermions renewed interest in semiconductor–superconductor interfaces, while a quest for higher-order non-Abelian excitations demands formation of superconducting contacts to materials with fractionalized excitations, such as a two-dimensional electron gas in a fractional quantum Hall regime. Here we report induced superconductivity in high-mobility two-dimensional electron gas in gallium arsenide heterostructures and development of highly transparent semiconductor–superconductor ohmic contacts. Supercurrent with characteristic temperature dependence of a ballistic junction has been observed across 0.6 μm, a regime previously achieved only in point contacts but essential to the formation of well separated non-Abelian states. High critical fields (>16 T) in NbN contacts enables investigation of an interplay between superconductivity and strongly correlated states in a two-dimensional electron gas at high magnetic fields.

## Abstract (web summary)

Superconductivity has been induced in 2D electron gases, but high-field interplay between it and quantum Hall edge states remains elusive. Here the authors reach this regime by growing transparent superconducting contacts in GaAs, reporting modification of resistance in the quantum Hall regime.

## Graphical abstract

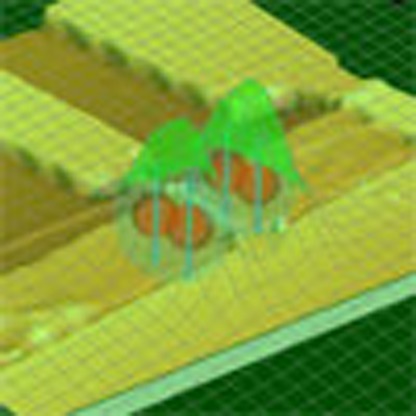

Introduction of Josephson field effect transistor concept^1^ sparked active research on proximity effects in semiconductors. Induced superconductivity and electrostatic control of critical current has been demonstrated in two-dimensional gases in InAs^2^,^3^, graphene^4^ and topological insulators^5^,^6^,^7^,^8^, and in one-dimensional systems^9^,^10^,^11^ including quantum spin Hall edges^12^,^13^. Recently, interest in superconductor–semiconductor interfaces was renewed by the search for Majorana fermions^14^,^15^, which were predicted to reside at the interface^16^,^17^,^18^. More exotic non-Abelian excitations, such as parafermions (fractional Majorana fermions)^19^,^20^,^21^ or Fibonacci fermions may be formed when fractional quantum Hall edge states interface with superconductivity. Realization of a long-sought regime of an interplay between superconductivity and strongly correlated states in a two-dimensional electron gas (2DEG) at high magnetic fields^22^,^23^,^24^,^25^,^26^,^27^ requires development of transparent superconducting contacts to high-mobility 2DEG, which remain superconducting at high magnetic fields.

Proximity effects in GaAs quantum wells have been intensively investigated in the past and Andreev reflection has been observed by several groups^28^,^29^,^30^,^31^. Unlike in InAs, where Fermi level (*E*_F_) at the surface resides in the conduction band, in GaAs *E*_F_ is pinned in the middle of the gap, which results in a high Schottky barrier between a 2DEG and a superconductor and low transparency non-ohmic contacts. Heavy doping can move *E*_F_ into the conduction band and, indeed, superconductivity has been induced in heavily doped bulk n^++^ GaAs^32^. In quantum wells, similar results were obtained by annealing indium contacts^33^; however, the critical field of indium is ∼30 mT, well below the fields required to form quantum Hall effect (QHE) states.

In this article, we report the development of transparent superconducting ohmic contacts to high-mobility 2DEG in GaAs. The superconducting contact is type-II NbN with large critical field >16 T. Induced superconductivity is observed across 1.6 μm of a 2D gas at zero field. From temperature dependence of the critical current and analysis of Andreev reflection, we estimate contact transparency parameter *Z*
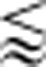
0.2. Induced superconductivity is observed in magnetic fields up to 0.2 T. At high magnetic fields, we observe deviations of longitudinal and Hall resistances from a similar data obtained with normal contacts, a clear indication of an interplay between superconductivity and QHE edge states.

## Results

### Heterostructures design

**Figure 1:**
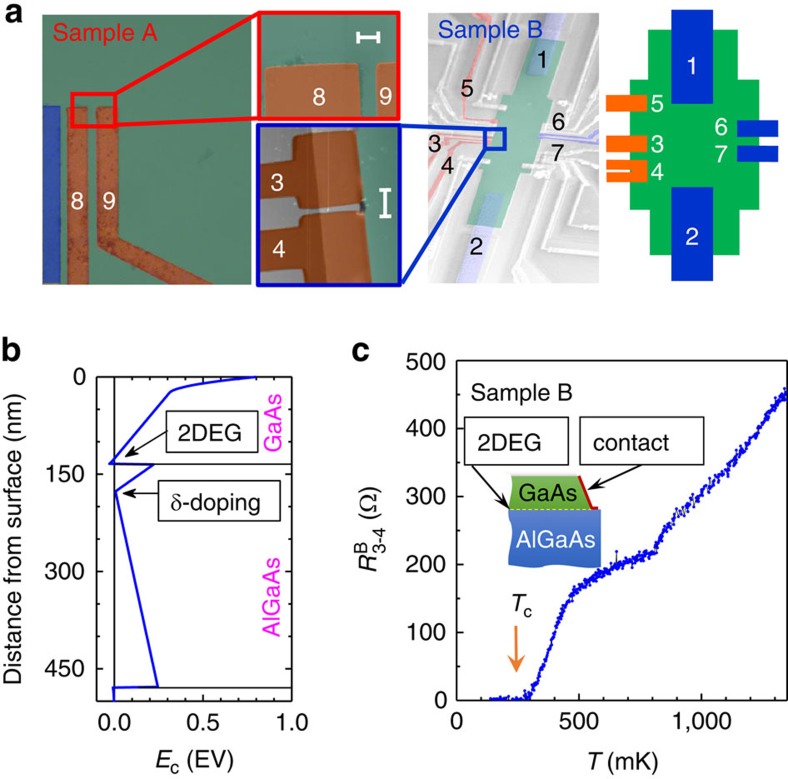
Design and superconducting transition. (**a**) Scanning electron microscope images of test devices similar to samples A and B. Enlarged region for sample B is an atomic force microscope image of a real sample. 2D gas regions are false-colour coded with green, superconducting and normal contacts are coded with orange and blue, respectively. Scale bar is 2 μm. (**b**) Simulation of the conduction band energy profile in the heterostructure. (**c**) *T*-dependence of resistance between contacts 3 and 4 in sample B measured with 10 nA a.c. excitation. Superconducting transition is observed at *T*_c_≈290 mK.

In conventional quantum well structures AlGaAs, barrier between 2DEG and the surface of the sample adds an extra 0.3 eV to the Schottky barrier when contacts are defused from the top. We alleviated these problems by growing an inverted heterojunction structures, where a 2DEG resides at the GaAs/AlGaAs interface but the AlGaAs barrier with modulation doping is placed below the 2DEG, see Fig. 1b, where band diagram was calculated using a self-consistent Poisson solver^34^ (The program can be downloaded from http://www3.nd.edu/~gsnider/). Contacts are recessed into the top GaAs layer to bring the superconductor closer to the 2DEG. A thin layer of AuGe and NbN superconductor form low resistance ohmic contacts to the 2DEG after annealing. The inverted heterostructure increases the contact area of side contacts compared with quantum well structures by utilizing all GaAs layer above the heterointerface for carrier injection (130 nm in our inverted heterostructure versus 20–30 nm in typical quantum wells, see Supplementary Fig. 1 and Supplementary Note 1).

### Induced superconductivity

We report induced superconductivity in two devices from different wafers. Sample A has long (70 μm) contacts separated by 1.6 μm of 2DEG, contacts to sample B are formed to the edge of a mesa with 0.6 μm separation, see Fig. 1a. Details of device fabrication are described in Methods. When cooled down to 4 K in the dark, both the samples show resistance in excess of 1 MΩ. After illumination with red light-emitting diode a 2DEG is formed and 2-terminal resistance drops to <500 Ω. As shown in Fig. 1d, sample resistance *RB*_3−4_ gradually decreases on cooldown from 4 K to the base temperature and the superconductor–2DEG–superconductor (S–2DEG–S) junction becomes superconducting at *T*_c_∼0.3 K.

**Figure 2:**
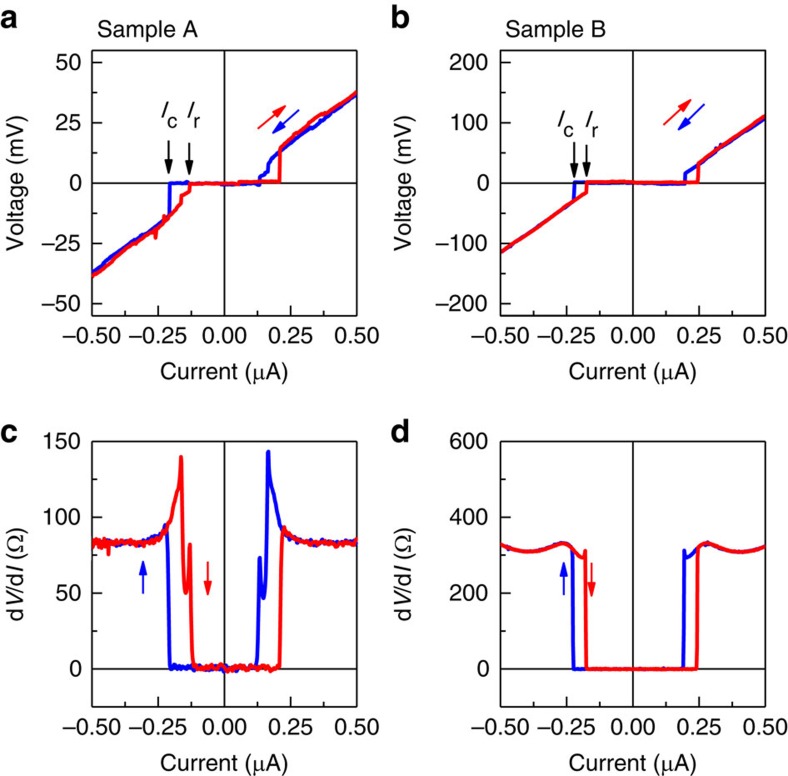
Induced superconductivity in a high-mobility 2D electron gas in GaAs. Voltage-current characteristics (**a**,**b**) and differential resistance (**c**,**d**) for samples A (**a**,**c**) and B (**b**,**d**). The conduction is measured between contacts (8-9) for sample A and (3-4) for sample B. d*V*/d*I* is measured with *I*_a.c._=1 nA. Induced superconductivity with zero voltage is observed with critical currents *I*_c_∼220 nA for sample A and *I*_c_∼230 for sample B. Red (blue) traces are for current increasing (decreasing).

Voltage–current *V*(*I*) characteristics for two S–2DEG–S junctions (between contacts 8 and 9 for sample A, and 3 and 4 for sample B) are shown in Fig. 2. Both the samples show zero-resistance state at small currents with abrupt switching into resistive state at critical currents *I*_c_=0.22 and 0.23 μA for samples A and B, respectively. We attribute hysteresis in *V*(*I*) characteristics to Joule heating in the normal state.

**Figure 3:**
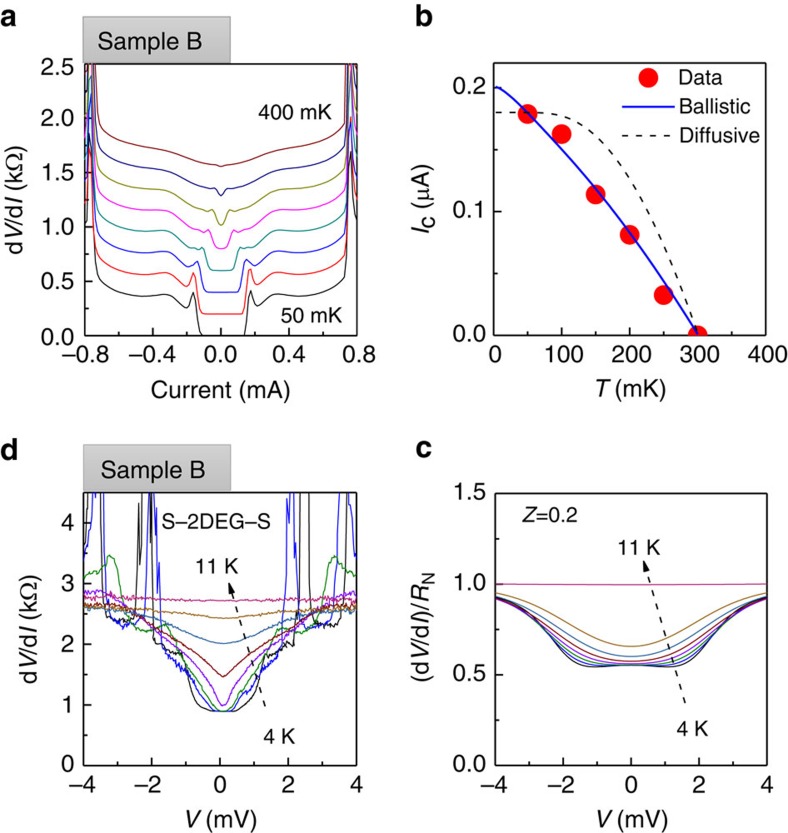
Temperature dependence of superconductivity in a ballistic junction. (**a**) Evolution of the induced superconductivity with *T* for sample B. The *R*(*I*) curves are offset proportional to *T* for *T*>50 mK. (**b**) Temperature dependence of critical current *I*_c_(*T*) is extracted from (**a**) and compared with the expected *T*-dependence for ballistic and diffusive regimes (reduced *I*_c_ compared with Fig. 2 is due to larger *I*_a.c._=10 nA used in this experiment). (**c**) High-temperature data shows Andreev reflection (excess current and reduced d*V*/d*I* around *V*=0. The curves are not offset. In **d**, excess current is modelled within the Blonder–Tinkham–Klapwijk theory^39^ with *Z*=0.2.

The most attractive property of a high-mobility 2DEG is large mean free path *l*≫*ξ*_0_, with *l*=24 μm and the Bardeen-Cooper-Schrieffer (BCS) coherence length *ξ*_0_=ℏ*v*_F_/*π*Δ=0.72 μm for sample B. Here 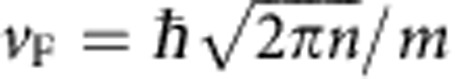 is the Fermi velocity, *n* is a 2D gas density, *m* is an effective mass and Δ=1.76*k*_B_*T*_c_=46 μeV is the induced superconducting gap. Evolution of *V*(*I*) with *T* is shown in Fig. 3a. Experimentally obtained *T*-dependence of *I*_c_ is best described by the Kulik–Omelyanchuk theory for ballistic junctions (*L*<<*l*) (ref. 35), the blue curve in Fig. 3b. For comparison, we also plot *I*_c_(*T*) dependence for the dirty limit 
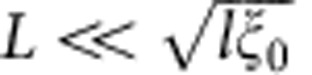
 (ref. 36), which exhibits characteristic saturation of *I*_c_ at low temperatures.

In short ballistic junctions, *L*<<*ξ*_0_<<*l* the product *I*_c_(0)*RN*=*π*Δ/*e* does not depend on the junction length *L*. For *L*∼*ξ*_0_ this product is reduced by a factor 2*ξ*_0_/(*L*+2*ξ*_0_) (ref. 37). The measured *I*_c_*RN*=83 μV for sample B is in a good agreement with an estimate *π*Δ/*e*·2*ξ*_0_/(*L*+2*ξ*_0_)=90 μV. For sample A, the *I*_c_*RN*=19 μV while the estimated product is ≈50 μV. The reduction is consistent with the geometry of sample A, where a region of the 2DEG with induced superconductivity is shunted by a large region of a 2DEG in a normal state.

### Transparency of a superconductor/2DEG interface

**Figure 4:**
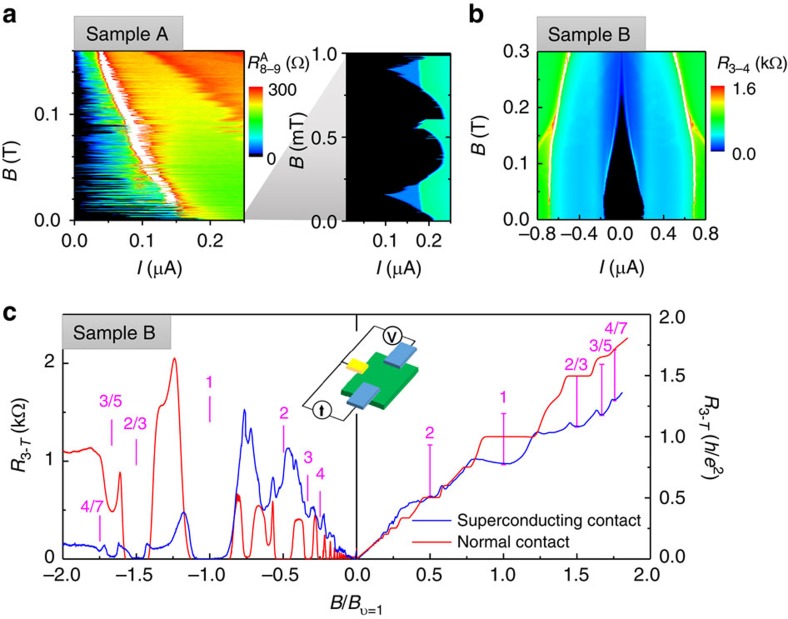
Magnetic field dependence of induced superconductivity. (**a**,**b**) Differential resistance is measured as a function of *B* and *I*_d.c._ for two samples at 40 mK. Induced superconductivity (black region) is observed up to 0.2 T in both the samples. (**c**) 3-terminal resistance for a sample with all normal contacts (red) and between normal and superconducting contacts in sample B (*I* (2–4) and *V* (4−1) in Fig. 1) is measured at 70 and 40 mK, respectively. *B*<0 (*B*>0) induces clockwise (counterclockwise) chiral edge channels, note resistance scales difference for two field directions.

In one-dimensional junctions, the induced gap 
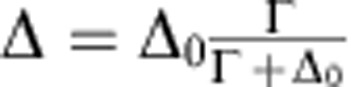
 depends on the broadening of Andreev levels within the semiconductor^38^ 
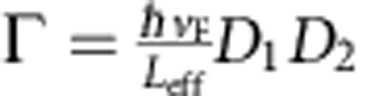
, where we introduce contacts transparencies *D*_1_ and *D*_2_. We assume for simplicity that *D*_1_=*D*_2_=1/(1+*Z*^2^), where 0<*Z*<∞ is a interface barrier strength introduced in ref. 39, and Bagwell's effective channel length *L*_eff_=*L*+2*ξ*_0_. Using NbN superconducting gap, 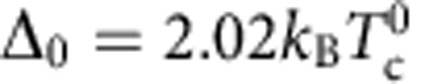 (NbN is a strong-coupling superconductor, 
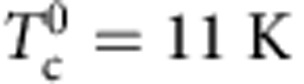
) and *T*_c_=0.3 K for 
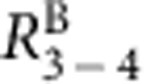
 we obtain *Z*=0.2. This value is consistent with the fit of the *I*_c_ versus *T*-dependence with *D* as a free parameter (Supplementary Fig. 2; Supplementary Note 2). Similar values of *Z* can be estimated from the analysis of the shape of d*I*/d*V*(*V*) characteristics at elevated temperatures, as shown in Fig. 3. At 
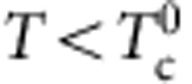
, Andreev reflection at S–2DEG interfaces results in an excess current flowing through the junction for voltage biases within the superconducting gap Δ_0_/*e* and corresponding reduction of a differential resistance d*V*/d*I* by a factor of 2. In the presence of a tunnelling barrier, normal reflection competes with Andreev reflection and reduced excess current near zero bias, resulting in a peak in differential resistance. Within the Blonder–Tinkham–Klapwijk theory^39^, a flat d*V*/d*I*(*V*) within Δ_0_/*e*, observed in our experiments, is expected only for contacts with very high transparency *Z*<0.2. For larger *Z*>0.2, a peak at low biases is expected (Supplementary Fig. 3, Supplementary Note 3). Several features of the experimental *I*(*V*) need to be mentioned. First, we observe several sharp peaks in the resistance at high biases (around 2 and 4 mV for *T*=4 K). Similar sharp resonances has been observed previously^40^, where authors attributed their appearance to the formation of Fabry–Pérot resonances between superconducting contacts. In our devices, the superconducting region is shunted by a low resistance (<100 Ω) 2DEG, thus appearance of >10 kΩ resonances cannot be explained by resonant electron trapping between contacts. These resonances are also observed in *I*(*V*) characteristics of a single S–2DEG interface (measured in the S–2DEG–N configuration between contacts 3 and 6, see Supplementary Fig. 3). Differential resistance does not change substantially across resonances, ruling out transport through a localized state. We speculate that in the contacts where these resonances are observed superconductivity is carried out by quasi-one-dimensional channels, and jumps in *I*/*V* characteristics are due to flux trapping at high currents. This scenario is consistent with the observation that peaks shift to lower currents at higher fields, see Fig. 4. The second notable feature of our data is reduction of the zero-bias resistance by ≈2.6 times at low temperatures, while Andreev reflection limits the reduction to the factor of 2. We attribute this reduction to the multiple Andreev reflection between two closely spaced contacts, for contacts with larger separation (20 μm) multiple Andreev reflection is suppressed and the reduction of resistance by a factor of 2 is observed (Supplementary Fig. 3).

### Induced superconductivity in low magnetic fields

Finally, we present magnetic field dependence of induced superconductivity. The low-field data is shown in Fig. 4a,b, where black regions correspond to zero-differential resistance. Induced superconductivity is suppressed at ≈0.2 T in both the samples. In sample A, a narrow region of a 2DEG with induced superconductivity is confined between large NbN superconducting leads with rigid phases. Perpendicular magnetic field twists the phase in the 2DEG resulting in Fraunhofer-like oscillations of the critical current. In this sample, although the 2DEG extends beyond the narrow region between the contacts and *I*_c_ does not decrease to zero and abrupt jumps in *I*_c_ reflect multiple flux jumps. The period of oscillations is ∼0.5 mT, which corresponds to an area of 4.1 μm^2^, much smaller than the area of the 2DEG between the contacts (≈120 μm^2^). This observation is consistent with the reduced *I*_c_*RN* product measured for this sample as discussed above. In sample B, contacts are fabricated along the edge of the mesa and 2D gas is not enclosed between the contacts. Consequently, *I*_c_ is a smooth function of *B*.

### Superconductivity and quantum Hall effect

Competition between superconductivity and chiral quantum Hall edge states is shown in Fig. 4c, where resistance is measured in a 3-terminal configuration over a wide range of magnetic fields. Simple Landauer–Büttiker model of edge states predicts zero resistance for negative and quantized Hall resistance for positive field direction for integer QHE and fractional QHE states, which is clearly seen in a sample with non-superconducting (AuGe) ohmic contacts (red curve). When a superconducting contact serves as a current injector (blue curve), integer *ν*=1 and fractional *ν*=2/3 and 3/5 states are well developed for *B*<0, while the same states are not quantized at proper QHE values for *B*>0. If we assume that current injection via a superconducting contact results in an extra voltage offset at the contact by *V*_off_≈Δ_ind_/*e*, the measured voltage will be reduced by *V*_off_. The magenta bars for *B*>0 indicate corrected resistance (*V*−*V*_off_)/*I* for *V*_off_=140 μV. While this offset may explain the measured values for fractional states, a twice smaller *V*_off_ is needed to reconcile the resistance at *ν*=1. Note that NbN critical field *B*_c_>16 T. At low fields, states *ν*=3, 4 and 5 have resistance minima for *B*<0, indicating a partial equilibration of chiral edge currents with the superconducting contact, while resistance near *ν*=2 has a maximum. Zero resistance at *ν*=1 and large resistance at *ν*=2 are in contrast to the theoretical prediction that *ν*=2 state should be stronger coupled with a superconducting contact than *ν*=1 (ref. 23).

## Methods

### GaAs wafers design and parameters

The GaAs/AlGaAs inverted heterojunctions were grown by molecular beam epitaxy on semi-insulating (100) GaAs substrates with the heterointerface placed 130 nm below the surface and *δ*-doping layer 30–40 nm below the GaAs/AlGaAs interface. Samples were fabricated from two wafers with density and mobility *n*=2.7 × 10^11^ cm^−2^, *μ*=2 × 10^6^ V s cm^−2^ (sample A) and *n*=1.7 × 10^11^ cm^−2^, *μ*=4 × 10^6^ V s cm^−2^ (sample B).

### Fabrications of superconducting contacts

Superconducting contacts were defined by standard electron beam lithography. First, a 120 nm—deep trench was created by wet etching. Next, samples were dipped into HCl:H_2_O (1:6) solution for 2 s and loaded into a thermal evaporation chamber, where Ti/AuGe (5/50 nm) was deposited. Finally, 70 nm of NbN was deposited by DC magnetron sputtering in Ar/N_2_ (85/15%) plasma at a total pressure of 2 mTorr. Deposition conditions were optimized for producing high quality NbN films with *T*_c_=11 K and *B*_c_>15 T, see Supplementary Fig. 4, and with minimal strain^41^. After metallization, contacts were annealed at 500 °C for 10 min in a forming gas (10% H_2_ in Ar). Measurements were performed in a dilution refrigerator with the base temperature <30 mK, high-temperature data was obtained in a variable temperature ^3^He system. Samples were illuminated with red light-emitting diode at 4 K to form a 2D gas, 2-terminal resistance drops from >1 MΩ before illumination to <500 Ω after illumination.

## Additional information

**How to cite this article:** Wan, Z. *et al*. Induced superconductivity in high-mobility two-dimensional electron gas in gallium arsenide heterostructures. *Nat. Commun.* 6:7426 doi: 10.1038/ncomms8426 (2015).

## Supplementary Material

Supplementary Information Supplementary Figures 1-4, Supplementary Note 1-3 and Supplementary References
