# Supplementary material for: Induced superconductivity in high-mobility two-dimensional electron gas in gallium arsenide heterostructures
Source: Nat Commun. 2015 Jun 11;6:7426. doi: 10.1038/ncomms8426 (PMC4490403; doi:10.1038/ncomms8426)
Supplement: Supplementary Information — Supplementary Figures 1-4, Supplementary Note 1-3 and Supplementary References [file ncomms8426-s1.pdf]

SUPPLEMENTARY FIGURES

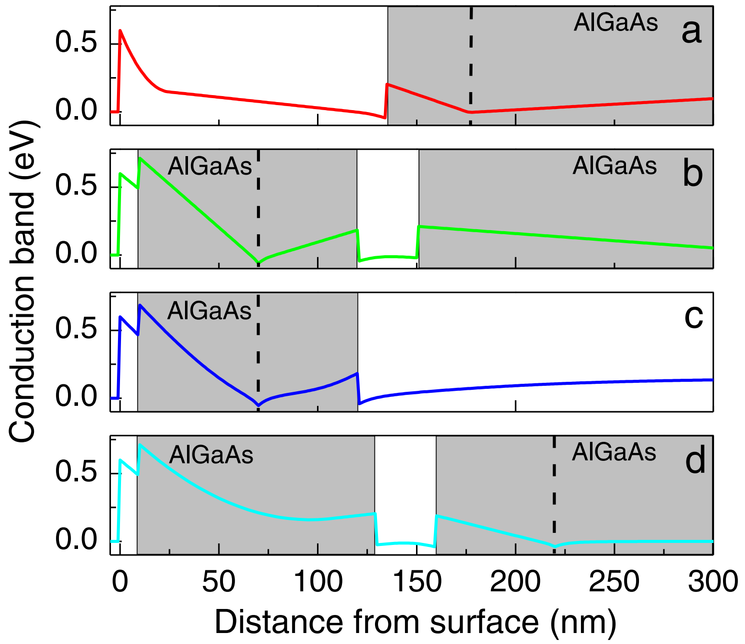

Supplementary Figure 1. **Comparison between conventional heterostructure and inverted single interface heterojunction.** Conduction band profile is plotted for (a) inverted single interface heterojunction used in our experiments and typical (b) modulation-doped quantum well, (c) single heterojunction, and (d) inverted quantum well. Dash lines indicate position of modulation doping.

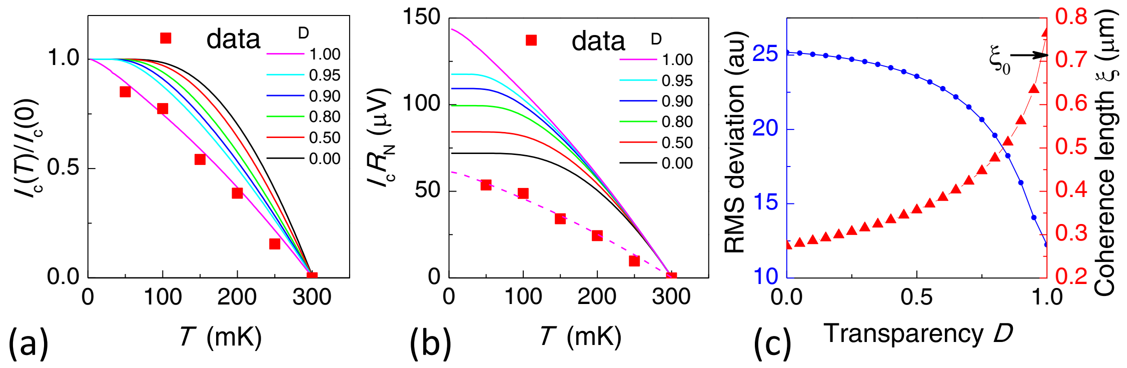

Supplementary Figure 2. **Analysis of the temperature dependence of the critical current.** Scaled (a) and unscaled (b) product I_cR_N is calculated using Eq. (1) for different transparencies D and $\alpha = 1$. Red dots are experimental data. Dashed line in (b) is for $\alpha = 0.7$ and $D = 1$. In (c) root-mean-square deviation between the best fit and the experimental data is shown for different D, coherence length ξ obtained from the best fit are red triangles.

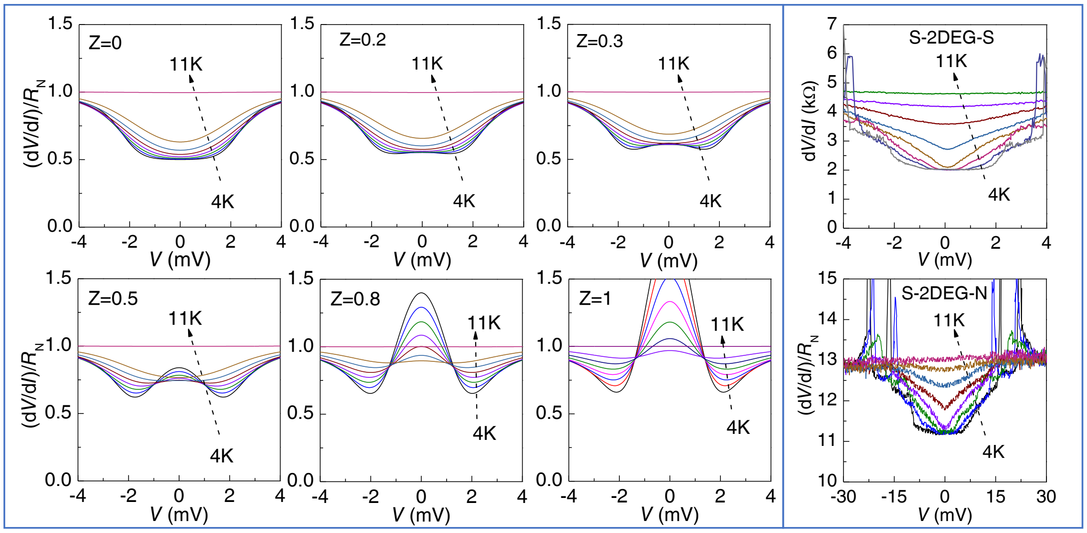

Supplementary Figure 3. **Temperature dependence of differential resistance.** Left 6 plots: normalized differential resistance is calculated using BKT theory, Eq. 2, for different barriers Z and temperatures between 4 and 11 K with a step of 1 K. Right 2 plots: experimentally measured differential resistance between two superconducting contacts (R_{3-5}) and a normal-superconducting contact (R_{4-7}) in sample B (the normal contact has high resistance).

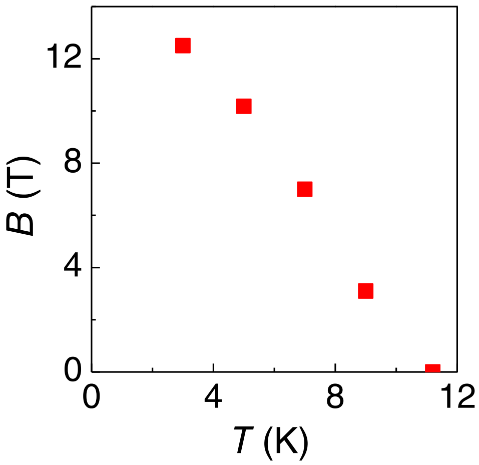

Supplementary Figure 4. **Temperature dependence of the NbN critical field.** Critical field of a 40 nm NbN film sputtered with optimal conditions is measured as a function of temperature.

SUPPLEMENTARY NOTES

Supplementary Note 1. Comparison between conventional heterostructures and an inverted single interface heterojunction

Comparison between conventional heterostructures and inverted single heterointerface structures used in this work is shown in Supplementary Figure 1. In conventional quantum well (b,d) and single interface heterostructures AlGaAs barrier between 2D gas and the surface adds 0.3 eV to the Schottky barrier if contacts are defused from the surface. For side contacts inverted single heterointerface (a) increases the exposed GaAs cross section for Cooper pair injection.

Supplementary Note 2. Temperature dependence of the critical current

Haberkorn et al. [1] generalized Kulik-Omelyanchuk current-phase relations[2, 3] to the case of arbitrary transparency of a tunnel barrier D inserted into the Josephson junction by directly solving Gor'kov's equations. They obtain the following current-phase relation:

$$I_{\mathrm{s}}(\phi, T)R_{\mathrm{N}} = \alpha \frac{\pi\Delta(T)}{2e} \frac{\sin(\phi)}{\sqrt{1 - D\sin^2(\phi/2)}} \times \tanh \frac{\Delta(T)}{2k_{\mathrm{B}}T} \sqrt{1 - D\sin^2(\phi/2)}, \tag{1}$$

where $\Delta(T)$ is the BCS gap. For $\alpha = 1$ this equation interpolates between diffusive ($D = 0$) and ballistic ($D = 1$) junctions. Critical current can be found as $I_{\mathrm{c}}(T)R_{\mathrm{N}} = max[I_{\mathrm{s}}(\phi, T)R_{\mathrm{N}}]$. We introduce coefficient α to account for the reduction of the critical current due to the finite length of the junction L, $\alpha = 2\xi/(L+2\xi)$ [4]. The best fit to the experimental $I_{\mathrm{c}}R_{\mathrm{N}}(T)$ dependence assuming both α and D as free parameters is obtained for $D = 1$ and $\alpha = 0.7$, see Fig. 2(a,b). For the contact spacing $L = 0.63$ μm this α corresponds to $\xi = 0.76$ μm, consistent with the BCS coherence length $\xi_0 = \hbar v_{\mathrm{F}}/\pi\Delta = 0.72$ μm. Transparency D can be related to the dimensionless barrier strength Z introduced in the Blonder-Tinkham-Klapwijk (BTK) theory[5], $D = 1/(1 + Z^2)$, and the fit sets the upper limit on Z, $Z < 0.1$. The quality of the fit parameters can be assessed from Fig. 2(c), where RMS error for the best fit with a fixed D and α as a free parameter (RMS deviation)^2 = $\sum_i \{[I_{\mathrm{c}}(T_i)R_{\mathrm{N}}]^{theory} - [I_{\mathrm{c}}(T_i)R_{\mathrm{N}}]^{exp}\}^2$ is plotted for different D. The RMS deviation has a clear global minimum at $D \to 1$. Note that the coherence length for $D < 1$, obtained from the fitting parameter α, becomes smaller than the estimated ξ_0.

Supplementary Note 3. Analysis of excess current above the induced superconductivity gap

Transparency of the superconductor-semiconductor interface can be estimated from the shape of the $dV/dI(V)$ characteristic, where competition between Andreev and normal reflections results in a peak in differential resistance when a tunneling barrier is present at the superconductor-semiconductor interface (transmission $D = 1/(1 + Z^2) < 1$). Differential resistance for different

temperatures can be calculated using Blonder-Tinkham-Klapwijk (BTK) theory[5]:

$$\frac{dI}{dV}(V) \propto \int_{-\infty}^{\infty} \frac{\partial f_0(E-eV)}{\partial(eV)}[1+A(E)-B(E)]dE, \tag{2}$$

where $f_0(E)$ is the Fermi Dirac function and $A(E)$ and $B(E)$ are energy-dependent Andreev and normal reflection coefficients, respectively. Both coefficients depend on the gap of NbN $\Delta_0 = \Delta(T)$ with $T_c^0 = 11$ K and the interface barrier strength Z. In Supplementary Figure. 3 we plot differential resistance for different values of Z. At low T for $Z = 0$ the barrier is transparent ($D = 1$) and all incident electrons are Andreev reflected, which leads to the a reduction of differential resistance by a factor of 2 within the energy gap Δ_0. When Z is finite, part of the incident electrons undergoes normal reflection which results in the increase of the resistance within the gap.

The exact shape of experimental curves differ from the shape predicted by the BKT theory, the most important deviation being sharp minima near $V = 0$ observed at T close to T_c^0 as compared to a much smoother BKT dependence. To account for a similar sharpening of a zero-bias peak in less transparent contacts ($Z > 2$) it has been assumed that a thin normal region is formed between NbN contacts and a 2DEG[6]. This more elaborate theory introduces two more fitting parameters for the superconducting-normal and normal-2DEG interfaces, but does not change the main qualitative prediction of a simpler BTK theory: appearance of a peak near $V = 0$ for $Z > 0.2$ in $dV/dI(V)$ characteristics.

Experimentally, we observe no zero-bias peak in $dV/dI(V)$ characteristics measured between two superconducting contacts R_{3-4} (S-2DEG-S) or between superconducting and normal contacts R_{8-9} (S-2DEG-N), see Figure. 3 and Supplementary Figure 3, thus we can set an upper limit $Z < 0.2$ and lower limit $D > 0.96$ for our contacts.

SUPPLEMENTARY REFERENCES

[1] W. Haberkorn, H. Knauer, and J. Richter, "A theoretical study of the current-phase relation in Josephson contacts," Physics Status Solidi **47**, K161–K164 (1978).

[2] I.O. Kulik and A.N. Omel'yanchuk, "Properties of superconducting microbridges in the pure limit." Soviet Journal of Low Temperature Physics **3**, 459 – 461 (1977).

[3] I.O. Kulik and A.N. Omel'yanchuk, "Microscopic theory of the Josephson effect in superconducting bridges," Pisma v ZETF **21**, 216 – 19 (1975).

[4] Philip F. Bagwell, "Suppression of the Josephson current through a narrow, mesoscopic, semiconductor channel by a single impurity," Phys. Rev. B **46**, 12573–12586 (1992).

[5] G. E. Blonder, M. Tinkham, and T. M. Klapwijk, "Transition from metallic to tunneling regimes in superconducting microconstrictions: Excess current, charge imbalance, and supercurrent conversion," Phys. Rev. B **25**, 4515–4532 (1982).

[6] K. Neurohr, A. A. Golubov, Th. Klocke, J. Kaufmann, Th. Schäpers, J. Appenzeller, D. Uhlisch, A. V. Ustinov, M. Hollfelder, H. Lüth, and A. I. Braginski, "Properties of lateral Nb contacts to a two-dimensional electron gas in an $In_{0.77}Ga_{0.23}As/InP$ heterostructure," Phys. Rev. B **54**, 17018–17028 (1996).
